# Drug-Induced Parkinsonism: A Structured, Mechanism-Informed Approach to Identification and Management

**DOI:** 10.7759/cureus.98340

**Published:** 2025-12-02

**Authors:** Beatriz De Faria Sousa, Juan B Espinosa

**Affiliations:** 1 College of Medicine, Florida International University, Herbert Wertheim College of Medicine, Miami, USA; 2 Department of Psychiatry, Florida International University, Miami, USA; 3 Department of Psychiatry, Memorial Regional Hospital, Hollywood, USA

**Keywords:** clinical reasoning, diagnostic framework, dopaminergic mechanisms, drug-induced parkinsonism, iatrogenic neurology, medical education, movement disorders, pharmacovigilance, polypharmacy, reversible parkinsonism

## Abstract

Drug-induced parkinsonism (DIP) is an underrecognized form of secondary parkinsonism that can mimic idiopathic Parkinson’s disease yet is largely reversible. While the use of first-generation antipsychotics has declined, additional agents, including valproate, cyclosporine, vesicular monoamine transporter 2 inhibitors, and Rauwolfia serpentina-based herbal supplements, have emerged as notable contributors. This narrative review synthesizes epidemiologic, mechanistic, and clinical data from 2009 to 2025 to provide a structured, mechanism-informed approach for clinicians. Across studies, DIP accounted for roughly 15-20% of secondary parkinsonism, disproportionately affecting older adults and women. Functional imaging with dopamine transporter single-photon emission computed tomography typically demonstrates preserved striatal uptake, aiding differentiation from neurodegenerative parkinsonism. Recovery usually occurs within six to 12 months of discontinuing the offending agent, though a minority may experience persistent symptoms. The proposed three-step approach emphasizes comprehensive medication and supplement review, recognition of characteristic clinical patterns, and timely withdrawal with follow-up to confirm reversibility. This framework supports safer prescribing practices, reinforces clinician education, and enhances detection of this preventable condition.

## Introduction and background

Drug-induced parkinsonism (DIP) is a reversible syndrome characterized by bradykinesia, rigidity, and tremor caused by pharmacologic agents. DIP remains a well-established example of iatrogenic neurology, an entirely man-made syndrome that closely mirrors one of the most common neurodegenerative disorders, idiopathic Parkinson’s disease (PD). Clinically, it presents with signs and symptoms indistinguishable from PD. Yet, unlike PD, the underlying pathology is functional, reversible, and preventable.

Although DIP was first described in the 1950s following reserpine use, its epidemiologic footprint remains consistent across decades [1,2]. Contemporary studies estimate that DIP represents up to one-fifth of parkinsonian cases worldwide, particularly among elderly and polypharmacy-exposed patients [3,4]. The persistence of this entity, despite decades of recognition, reflects an enduring educational and diagnostic gap rather than a pharmacologic inevitability. Understanding the historical context of DIP provides a foundation for examining the contemporary epidemiologic and mechanistic landscape.

In the mid-20th century, typical antipsychotics such as chlorpromazine and haloperidol dominated the etiologic landscape. The subsequent transition to atypical agents, perceived as safer, was accompanied by a decline in reported cases but not their elimination [2]. Instead, newer pharmacologic classes have emerged as unexpected offenders. Valproate, widely used for mood stabilization and epilepsy, and cyclosporine, an immunosuppressant, have both been implicated in reversible parkinsonism [3]. Similarly, calcium channel antagonists such as cinnarizine and flunarizine, used for vertigo and migraine prophylaxis, have been identified in population analyses as notable culprits [5]. These pharmacologic shifts underscore the need to examine newer causative agents and evolving risk factors in the current era.

More recently, herbal formulations containing *Rauwolfia serpentina*, a natural source of the alkaloid reserpine, have reintroduced an ostensibly “ancient” mechanism of parkinsonism to modern practice [6]. These products, available without prescription, are frequently marketed for hypertension or anxiety. Their unregulated use illustrates the intersection of neuropharmacology, public health, and cultural medicine.

DIP is thus no longer confined to psychiatric pharmacotherapy; it now spans multiple clinical specialties. Understanding its epidemiology, converging pathophysiologic mechanisms, and evolving pharmacologic risk factors is crucial for accurate diagnosis and prevention. This narrative review synthesizes two decades of literature to summarize contemporary epidemiology, pharmacologic risk factors, mechanistic insights, and emerging diagnostic strategies for DIP, providing an accessible framework for clinicians and educators across disciplines.

## Review

Global epidemiology: expanding drug spectrum

Methods: Literature Selection Approach 

A narrative review was conducted on studies published between 2009 and 2025 using PubMed, Google Scholar, and WHO pharmacovigilance resources. Search terms included “drug-induced parkinsonism”, “secondary parkinsonism”, “dopamine receptor blockade”, “VMAT2 inhibitors”, and “valproate parkinsonism”. Population-based studies, pharmacovigilance analyses, mechanistic investigations, and relevant case reports were included based on their relevance to epidemiology, mechanisms, or diagnostic differentiation of DIP. As this is a narrative rather than a systematic review, no formal bias assessment or predefined inclusion criteria were applied. While not replicable in a strict Preferred Reporting Items for Systematic Reviews and Meta-Analyses (PRISMA) sense, this approach emphasizes conceptual reproducibility and allows integration of mechanistic and clinical insights not easily captured in traditional systematic reviews. Given the heterogeneity of included studies, no inferential statistical analyses or formal meta-analytic pooling were performed, and data are presented descriptively.

Population Studies: Epidemiology and Risk

Epidemiologic estimates of DIP vary widely, largely reflecting regional differences in prescribing habits. Savica et al., in a 30-year population-based study from Minnesota, identified 108 drug-induced cases among 906 total parkinsonism diagnoses (11.9%), corresponding to an incidence of 3.3 per 100,000 person-years [2]. The investigators noted a 68% decline in overall DIP incidence over three decades, primarily due to reduced typical antipsychotic use [2]. However, this apparent improvement was offset by increased reports linked to antidepressants and calcium channel blockers.

These data mirror earlier findings by Thanvi and Treadwell, who identified DIP as the second most common parkinsonian disorder among older adults [1]. Both studies converge on two key epidemiologic constants: age and sex. Women and individuals over 65 years old remain disproportionately affected, likely due to pharmacokinetic differences, receptor sensitivity, and the higher prevalence of polypharmacy [1].

Pharmacovigilance: Real-World Data

While population studies capture clinically confirmed cases, pharmacovigilance databases reveal a broader global footprint. de Germay et al. analyzed over 5,000 cases in WHO’s VigiBase and found that 71% involved antipsychotics, but 29% stemmed from nonpsychiatric drugs, including calcium channel blockers, antidepressants, immunosuppressants, and mood stabilizers [7]. Time to onset averaged five weeks, while median recovery occurred within six months of withdrawal. Notably, spontaneous recovery was less common in older adults and women, aligning with previous population trends [7].

Similarly, Bondon-Guitton et al. documented 193 cases across 17 years in France’s regional pharmacovigilance system [4]. Nearly half were attributed to dopamine antagonists, while the remainder involved cardiovascular or gastrointestinal medications. The authors emphasized that polypharmacy, age, and delayed recognition were consistent risk amplifiers.

Regional Patterns: Emerging Offenders

Data from Asia further underscore evolving patterns. Kim et al. conducted a population-based case-control study in Korea, identifying significant associations between DIP and chronic exposure to gastrointestinal prokinetics, antipsychotics, and calcium channel blockers [8]. Their analysis also revealed an underrecognized risk among valproate users, adding weight to previous mechanistic evidence linking valproate to mitochondrial dysfunction [3,8]. Notably, anticonvulsants beyond valproate, including pregabalin, have also been associated with reversible parkinsonism, possibly via GABAergic modulation that alters basal ganglia signaling in susceptible individuals [1,3,9].

Newer pharmacologic classes, including calcium channel blockers, valproate, and certain antidepressants such as escitalopram, have been implicated in DIP [10]. For calcium channel blockers and valproate, multiple population-based and mechanistic studies support their association with parkinsonism, likely via dopaminergic or mitochondrial effects. Reports linking antidepressants are less frequent and largely anecdotal; these findings should be interpreted with prudence, acknowledging the limited and preliminary nature of the evidence. The pharmacologic expansion of DIP reflects not only new drugs but also evolving prescribing patterns and increased awareness of subtle, reversible parkinsonism. This underscores the importance of mechanism-based understanding rather than reliance on historical lists of “culprit drugs.”

Mechanistic convergence: from synapse to syndrome

DIP exemplifies the principle that diverse molecular insults can produce a unified clinical phenotype. Despite distinct pharmacologic origins, nearly all causative agents disrupt dopaminergic signaling within the basal ganglia. Four major mechanistic pathways have been delineated:

D₂-Receptor Blockade

Classic neuroleptics (haloperidol and chlorpromazine) and antiemetics (metoclopramide and prochlorperazine) antagonize postsynaptic D₂ receptors in the striatum. The resultant decrease in dopaminergic tone manifests as bradykinesia and rigidity. Factor et al. note that even “atypical” antipsychotics such as risperidone can produce DIP at higher doses, particularly in susceptible individuals [5].

Vesicular Monoamine Transporter 2 (VMAT2) Inhibition

Reserpine, tetrabenazine, and deutetrabenazine deplete dopamine stores by irreversibly inhibiting the VMAT2 [11]. Rijntjes and Meyer reported a striking case of *R. serpentina*-induced parkinsonism from an over-the-counter herbal supplement, confirmed by normal dopamine-transporter imaging and full recovery post-withdrawal [6].

Mitochondrial and Oxidative Toxicity

Valproate and cyclosporine are suggested to impair mitochondrial oxidative phosphorylation, reducing ATP generation and increasing reactive oxygen species within basal ganglia neurons [3,12]. This mechanism blurs the line between “functional” and “neurotoxic” DIP, as recovery may be incomplete due to structural damage.

Calcium Channel Blockade and Metal Injury

Agents such as flunarizine and cinnarizine block T-type calcium channels in the striatum, altering neuronal excitability, and chronic exposure has been linked to parkinsonism in epidemiologic studies [8]. Similarly, heavy-metal exposure (e.g., manganese and iron) has been reported in case series and environmental studies to induce parkinsonian features via oxidative stress pathways, though evidence remains limited and causality is suggestive rather than definitive [5].

These mechanisms converge on dopaminergic insufficiency, producing a phenotype indistinguishable from PD. However, unlike PD, the presynaptic terminals remain structurally intact, an insight confirmed by modern imaging.

Integration: mechanistic and epidemiologic framework

Understanding DIP requires moving beyond causality lists toward mechanistic reasoning. As Liotta et al. described, cumulative pharmacologic effects can collectively depress dopaminergic tone, even if individual agents are subthreshold in risk [13]. The coexistence of multiple mechanisms (e.g., a patient taking valproate and an atypical antipsychotic) can precipitate DIP via additive pathophysiologic load [13].

López-Sendón et al. and Factor et al. both emphasize that such mechanistic overlap explains the clinical heterogeneity seen in DIP [3,5]. For instance, patients with valproate-induced parkinsonism often exhibit tremor-dominant phenotypes and slower recovery than those exposed to D₂ antagonists. Likewise, reserpine-induced cases frequently present with coexistent depression, reflecting its depletion of serotonin and norepinephrine alongside dopamine [6].

From a systems perspective, integrating pharmacovigilance and mechanistic insights reveals an opportunity: a diagnostic framework grounded not in memorization, but in neurobiology.

Recent advances further enrich this understanding. Functional imaging studies, including dopamine-transporter single-photon emission computed tomography (DAT-SPECT) and cardiac metaiodobenzylguanidine (MIBG) scintigraphy, confirm preserved presynaptic integrity in DIP and help distinguish it from idiopathic PD [3,6]. Pharmacogenomic investigations highlight how individual genetic variation in dopamine receptor sensitivity and drug metabolism may modulate DIP risk, particularly in older adults and women [1,3,5]. Finally, systems-level approaches focusing on deprescribing, medication reconciliation, and multidisciplinary monitoring demonstrate potential for preventing DIP in high-risk populations [2,4,7]. Together, these findings support a mechanism-guided framework that is clinically actionable, biologically informed, and preventive. They emphasize a systems approach, linking drug mechanisms to clinical recognition, educational opportunities, and patient safety. The next section translates this concept into clinical recognition and management principles.

Clinical Recognition: Pattern, Pitfalls, and Polypharmacy

The clinical portrait of DIP overlaps so closely with idiopathic PD that even experienced neurologists may falter in distinction. Symptoms typically develop subacutely, within weeks to months after exposure to the offending agent, and characteristically present symmetrically. Rigidity and bradykinesia dominate, while rest tremor, when present, is usually mild or postural rather than rhythmic [1,3]. Recent psychiatric case reports illustrate that DIP can mimic idiopathic PD both clinically and radiologically [14].

The absence of prodromal nonmotor symptoms, such as anosmia, constipation, rapid eye movement (REM) behavior disorder, or autonomic dysfunction, should raise suspicion for a drug-induced cause [5,6]. In contrast to PD, where early hyposmia or REM disturbance often precedes motor onset by years, DIP emerges abruptly following pharmacologic intervention [3].

Several risk amplifiers recur across studies: advanced age, female sex, and polypharmacy [2,4,7]. Older adults accumulate higher plasma concentrations due to reduced hepatic metabolism and renal clearance, while women demonstrate greater dopamine-receptor sensitivity [3]. The interaction of multiple agents, each with mild dopaminergic effects, can cumulatively exceed the threshold for dopaminergic dysfunction [13].

Psychiatric and gastrointestinal populations are most affected. In long-term antipsychotic therapy, for example, up to 60% of chronic schizophrenia patients develop parkinsonian features at some point, many fulfilling clinical PD criteria [5]. Similarly, metoclopramide and prochlorperazine, prescribed for nausea or gastroparesis, remain common iatrogenic triggers, particularly in elderly women.

Failure to recognize DIP leads to misdiagnosis and inappropriate levodopa treatment, which typically offers minimal benefit and may further strengthen the incorrect assumption of refractory PD. In a French series, 26% of presumed PD patients were ultimately reclassified as DIP after careful medication review [4].

Diagnostic imaging: DAT-SPECT and biomarkers

The cornerstone of diagnosis remains clinical suspicion coupled with systematic medication review. However, functional imaging now provides confirmatory evidence distinguishing functional dopaminergic blockade from true neurodegeneration.

DAT-SPECT Imaging

DAT-SPECT visualizes presynaptic transporter integrity. In PD, nigrostriatal degeneration causes an asymmetric reduction in striatal uptake, most prominent in the posterior putamen. In DIP, by contrast, DAT-SPECT demonstrates normal or symmetric uptake, reflecting preserved presynaptic terminals [3,6].

Rijntjes and Meyer described a patient who developed parkinsonism after taking a *R. serpentina *herbal supplement [6]; DAT-SPECT revealed intact uptake, and the patient recovered completely within weeks of discontinuation. Similar imaging patterns have been reported in valproate- and flunarizine-related cases [3,8].

Beyond its diagnostic value, DAT-SPECT has pedagogical significance. Visual confirmation of preserved dopaminergic terminals reinforces the conceptual distinction between functional inhibition and degenerative loss, an insight crucial for trainees learning to approach parkinsonism mechanistically.

Other Imaging and Biomarkers

Complementary tools can refine differentiation. Cardiac MIBG scintigraphy, assessing sympathetic innervation, is typically abnormal in PD but normal in DIP. Likewise, olfactory testing tends to show preserved function in DIP [3]. While not universally available, these modalities underscore a pattern of functional integrity amid pharmacologic blockade, the defining hallmark of the syndrome.

Unified framework: mechanism-driven diagnosis

The diversity of mechanisms and clinical presentations calls for a practical schema that clinicians can readily apply. Synthesizing evidence across epidemiology, neurobiology, and imaging, we propose a mechanism-guided diagnostic framework that operationalizes the recognition and management of DIP. Table 1 summarizes common pharmacologic agents known to precipitate or exacerbate parkinsonian symptoms, categorized by their principal mechanism of action. Dopamine-blocking, dopamine-depleting, and mitochondrial-toxic agents are highlighted as the most frequent contributors to DIP and form the basis for the diagnostic workflow discussed in the subsequent section.

**Table 1:** Mechanistic and diagnostic framework for DIP Representative drug classes and examples associated with DIP. Drug categories include dopamine-blocking agents (e.g., antipsychotics and metoclopramide), dopamine-depleting agents (e.g., reserpine and tetrabenazine), and mitochondrial-toxic drugs (e.g., valproate, cyclosporine, and lithium). These mechanistic groupings guide the clinical approach to identifying, differentiating, and managing DIP described in the text. DAT-SPECT, dopamine-transporter single-photon emission computed tomography; DIP, drug-induced parkinsonism; VMAT2, vesicular monoamine transporter 2

Mechanistic category	Representative agents	Proposed pathophysiology	Key clinical clues	Reversibility/prognosis	Diagnostic and management pearls
D₂-receptor blockade	Typical and atypical antipsychotics (haloperidol and risperidone); antiemetics (metoclopramide)	Postsynaptic D₂ antagonism → decreased striatal dopamine transmission	Symmetric bradykinesia > tremor; subacute onset	Recovery in six to 12 months after withdrawal [2,3]	DAT-SPECT normal; substitute with quetiapine/clozapine if psychiatric therapy required
VMAT2 inhibition	Reserpine, tetrabenazine, deutetrabenazine, *Rauwolfia serpentina*	Irreversible vesicular monoamine transporter inhibition → dopamine depletion	Parkinsonism ± depression, hypotension, fatigue	Reversible within months [6]	Ask about herbal use; DAT-SPECT preserved; avoid serotonergic co-medications
Mitochondrial/metabolic toxicity	Valproate, cyclosporine, lithium	Mitochondrial dysfunction and oxidative stress in the basal ganglia	Tremor-dominant; incomplete recovery	Persistent symptoms ≈ 15% [3,5]	Monitor hepatic and renal function; gradual improvement with amantadine support
Calcium channel/metal toxicity	Cinnarizine, flunarizine, manganese, and iron overload	T-type Ca²⁺ blockade and oxidative injury to dopaminergic neurons	Tremor > rigidity; dystonic features possible	Variable; may be incomplete [5,8]	Review vascular/vertigo drugs; stop agent; consider chelation if metal exposure
Polypharmacy/additive effects	Combination of mild D₂ or mitochondrial agents [13]	Cumulative dopaminergic stress from multiple low-risk drugs	Subtle, fluctuating parkinsonism	Gradual recovery after medication rationalization	Prioritize deprescribing; educate multidisciplinary teams

Operationalization: three-step diagnostic pathway

Building upon Table 1, the diagnostic reasoning process can be integrated into the bedside workflow through three sequential considerations. First, clinicians should conduct a comprehensive medication and supplement review, including all prescription, over-the-counter, and herbal exposures within the past 12 months. Particular attention should be given to dopamine-blocking agents (e.g., antipsychotics and metoclopramide), dopamine-depleting drugs (e.g., reserpine and tetrabenazine), and mitochondrial-toxic compounds (e.g., valproate, cyclosporine, and lithium), while also considering additive interactions between agents from different mechanistic categories.

Next, recognition and differentiation of DIP rely on identifying characteristic features such as symmetric rigidity and bradykinesia greater than tremor, with minimal prodromal nonmotor symptoms. A DAT-SPECT scan demonstrating preserved, symmetric striatal uptake supports a diagnosis of DIP, whereas abnormal or asymmetric uptake should prompt evaluation for idiopathic PD or another neurodegenerative etiology.

**Figure 1:**
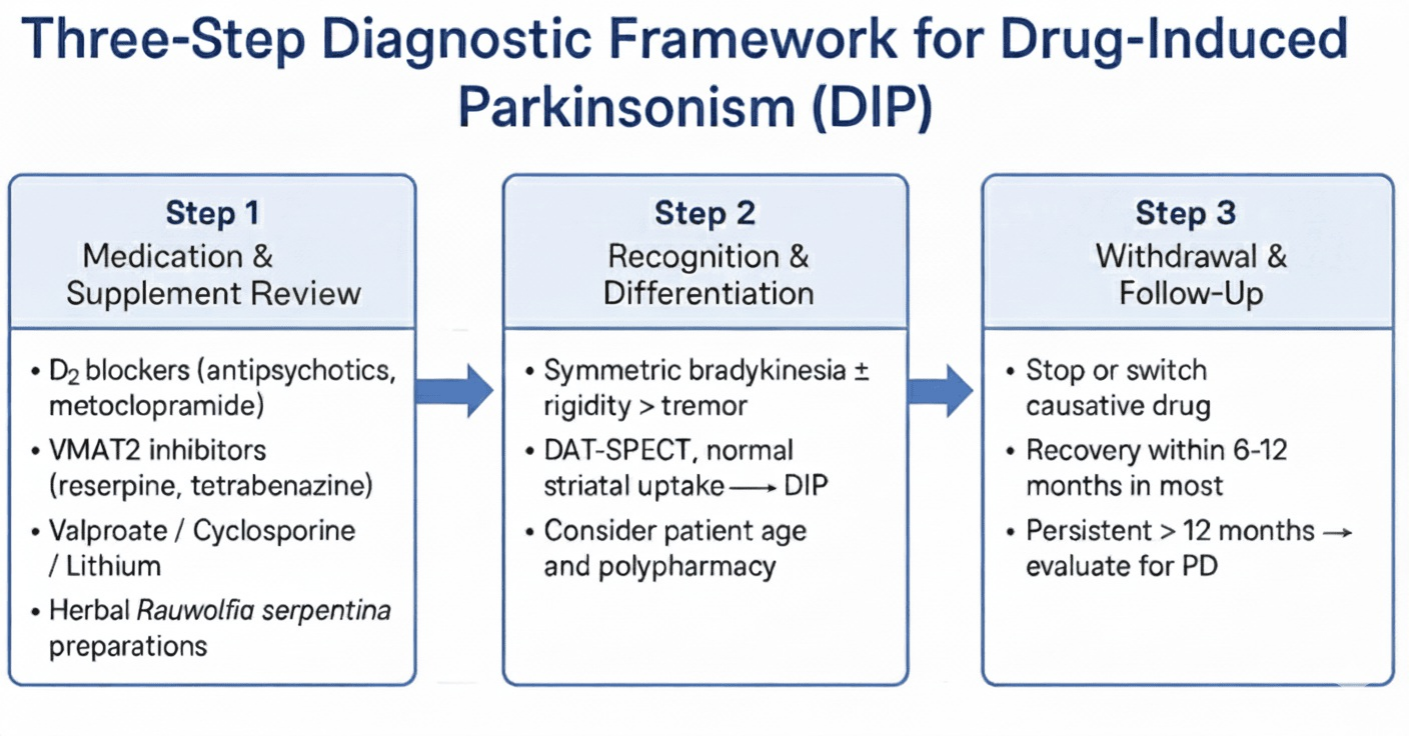
Three-step diagnostic framework for DIP Clinical algorithm for diagnostic evaluation of DIP. DAT-SPECT, dopamine-transporter single-photon emission computed tomography; DIP, drug-induced parkinsonism; PD, Parkinson’s disease; VMAT2, vesicular monoamine transporter 2 Created by Beatriz De Faria Sousa

Finally, management involves gradual discontinuation or substitution of the offending drug, followed by clinical monitoring for six to 12 months. Approximately 85-90% of patients experience full recovery [2,3]. Persistence of symptoms beyond 12 months suggests unmasking of PD or mitochondrial injury [15]. Patient and prescriber education on avoiding reexposure is essential for long-term prevention. Figure 1 illustrates the three-step diagnostic framework described above.

Therapeutic principles and prognosis

Drug Withdrawal and Substitution

Drug discontinuation remains the definitive treatment. In López-Sendón et al.’s review, symptom resolution occurred in 85-90% within one year, though the pace of recovery varied by mechanism [3]. D₂ blockade-related cases resolved most rapidly, while valproate- or cyclosporine-associated cases improved slowly and sometimes incompletely due to mitochondrial injury [15].

Gradual tapering is recommended for psychiatric medications to avoid withdrawal syndromes. When antipsychotic therapy is essential, quetiapine or clozapine is preferred due to lower D₂ affinity [5]. For gastrointestinal motility disorders, domperidone, which poorly crosses the blood-brain barrier, can replace metoclopramide [1].

Symptomatic and Supportive Therapy

Symptomatic treatments include amantadine, anticholinergics (e.g., trihexyphenidyl), and physical therapy. Amantadine enhances dopaminergic release and exerts NMDA-antagonist effects that may improve bradykinesia. Anticholinergics can reduce rigidity but risk cognitive side effects in older adults.

Evidence for dopaminergic therapy (levodopa) in DIP remains limited. In pure DIP, presynaptic dopamine terminals and transporter integrity are preserved, so levodopa generally provides minimal symptomatic relief. Consequently, levodopa is primarily used for diagnostic purposes: a positive response may indicate coexistent or unmasked idiopathic PD rather than true DIP. In cases where DIP overlaps with underlying neurodegeneration, levodopa may offer palliative benefit, but this is the exception rather than the rule. Clinicians should therefore view levodopa responsiveness as a tool to differentiate pathophysiology, not as a standard therapeutic strategy for pure DIP [3].

Prognostic Spectrum

The natural course of DIP spans from rapid remission to prolonged disability. Savica et al. reported a median recovery of six months after withdrawal [2]; Randhawa and Mehanna described persistence beyond 22 months in 15% of cases, particularly those linked to mitochondrial or oxidative mechanisms [15]. Reexposure to the same or similar agent predictably precipitates relapse, emphasizing the need for permanent avoidance documentation in medical records.

Functional and Psychosocial Consequences

Even transient parkinsonism carries a significant psychosocial impact: reduced mobility, stigma, and depression. Factor et al. underscore the importance of early identification and counseling to mitigate these burdens [5]. For patients whose DIP results from psychiatric therapy, coordinated care between neurologists and psychiatrists ensures that mental health treatment continues safely without reinstating parkinsonian risk.

Educational, Ethical, and System-Level Implications

DIP exemplifies how neurological syndromes can arise not from pathology but from pharmacology. Its persistence as a cause of morbidity reflects not a therapeutic inevitability but an educational gap. Across neurology curricula, emphasis remains on degenerative disorders such as PD, while iatrogenic syndromes receive minimal attention. Yet DIP is both common and preventable, an ideal teaching tool for linking neurobiology, clinical reasoning, and patient safety.

López-Sendón et al. noted that up to 40% of DIP patients initially receive dopaminergic therapy, a direct marker of diagnostic failure [3]. Despite abundant mechanistic data, misclassification persists across specialties, particularly psychiatry, geriatrics, and primary care. Factor et al. attribute this to “pattern-recognition bias,” in which clinicians, anchored by the classic motor triad of PD, overlook pharmacologic causes [5].

Educationally, DIP can serve as a pedagogic bridge between basic pharmacology and clinical medicine. Teaching students and residents to ask, “Could this be drug-induced?” before diagnosing PD cultivates diagnostic humility and prevents harm. Case-based teaching with real DAT-SPECT images reinforces how receptor blockade or VMAT2 inhibition alters function without structural loss [6,8].

Ethically, DIP raises issues of nonmaleficence and informed consent. When prescribing dopamine-blocking drugs, clinicians must weigh therapeutic benefit against neurologic risk, particularly in vulnerable populations. Informed discussions should include the potential for reversible parkinsonism. For those already affected, timely recognition honors autonomy and prevents unnecessary exposure to chronic PD therapies.

Systemic contributors and preventive strategies

The persistence of DIP reflects not only pharmacologic risk but also broader systemic vulnerabilities in contemporary healthcare. As Liotta et al. [13] noted, the polypharmacy-driven etiology of DIP often arises when multiple prescribers manage the same patient without coordinated oversight, leading to cumulative dopaminergic effects that may go unrecognized. In elderly populations, where polypharmacy is the rule rather than the exception, drug interactions that cumulatively impair dopaminergic signaling often go unnoticed. Electronic medical records rarely flag additive dopamine-receptor antagonism across specialties, leaving these iatrogenic risks unmitigated. Implementing medication review alerts for high-risk pharmacologic combinations could therefore prevent many cases before symptom onset, particularly in settings where multiple specialists prescribe concurrently.

Pharmacovigilance data, traditionally viewed as retrospective, can also be leveraged prospectively to strengthen prevention. Analyses from WHO VigiBase have highlighted underreported culprits such as calcium channel blockers and valproate [8]. Integrating these data into continuing medical education programs, prescribing software, and clinical decision support tools would transform passive surveillance into active prevention. When combined with electronic health record (EHR)-based algorithms, pharmacovigilance systems could provide real-time alerts for potentially harmful drug combinations, bridging the gap between population-level data and point-of-care decision-making.

The geriatric and psychiatric populations, described by Thanvi and Treadwell [1] as the “silent epicenter” of DIP, warrant particular vigilance, as subtle motor slowing is often misattributed to normal aging or psychiatric side effects. Routine neurologic screening during chronic pharmacotherapy, using simple bedside assessments such as finger tapping or rapid alternating movements during medication renewals, can identify emerging parkinsonism early. Ultimately, sustainable prevention requires interdisciplinary collaboration among neurology, psychiatry, primary care, and pharmacy. Embedding pharmacists within neurology clinics can facilitate continuous medication audits while establishing automatic neurology consultations for psychiatric inpatients after six months of antipsychotic therapy, which could detect early DIP before it progresses to irreversible disability.

Broader discussion: integrating mechanism, epidemiology, and education

DIP remains a prevalent, preventable condition, affecting approximately 11-20% of parkinsonism cases worldwide [2,8]. While causative agents evolve from antipsychotics to valproate and immunosuppressants, older, polypharmacy-exposed populations remain consistently vulnerable [8].

DIP arises from converging mechanisms, such as D₂-receptor blockade, VMAT2 inhibition, and mitochondrial or oxidative stress, culminating in bradykinesia and rigidity despite preserved presynaptic dopamine function, as confirmed by DAT-SPECT and cardiac MIBG imaging [3,6]. This mechanistic understanding informs prognosis: D₂-blockade-related cases resolve more rapidly, whereas recovery may be slower in mitochondrial or oxidative forms, though causality remains suggestive rather than definitive [15].

Management strategies are aligned to mechanism: select low-D₂-affinity antipsychotics, discontinue VMAT2 inhibitors with careful co-medication review, monitor metabolic and hepatic parameters in suspected mitochondrial dysfunction, and address metal or calcium-related exposures when relevant. Integrating mechanistic insight with epidemiology and imaging provides a concise, teachable framework, reinforcing patient safety, clinical reasoning, and educational value.

Future research and educational priorities

Future work in DIP must bridge genetics, digital surveillance, and education to prevent misdiagnosis and improve patient outcomes. At the molecular level, pharmacogenomic research should explore whether variations in genes such as VMAT2, DRD2, and mitochondrial enzymes confer differential susceptibility to DIP. Such genetic diversity could explain why some patients develop parkinsonism after minimal drug exposure while others tolerate prolonged therapy without symptoms. Kim et al. [8] proposed that calcium channel polymorphisms may influence recovery trajectories across populations, suggesting that personalized pharmacogenomic screening could one day stratify risk for patients requiring long-term dopaminergic antagonists.

On the systems and technological front, advances in pharmacovigilance and EHRs offer promising opportunities for prevention. Integrating data from WHO VigiBase and national surveillance networks directly into EHR systems could allow real-time alerts when high-risk drug combinations are prescribed, transforming reactive case reporting into proactive clinical safety. de Germay et al. [7] outlined this framework conceptually, envisioning interdisciplinary collaboration between neurology and pharmacy to pilot automated medication safety networks that prevent dopaminergic toxicity before symptom onset. Parallel efforts should extend beyond detection toward longitudinal understanding: neuroimaging studies that combine DAT-SPECT, MRI, and metabolic PET could clarify whether persistent cases reflect reversible synaptic dysfunction or evolving neurodegeneration while also identifying biomarkers predictive of recovery versus progression.

In addition, future research should prioritize the development and validation of diagnostic tools capable of distinguishing DIP from early PD. Studies evaluating the sensitivity and specificity of bedside assessments, imaging markers, and pharmacologic risk scores may help establish objective criteria that improve diagnostic accuracy in clinical settings. A formal systematic review or meta-analysis synthesizing quantitative epidemiologic and imaging data across studies could further clarify prevalence patterns, risk predictors, and recovery trajectories, enriching the evidence base for mechanism-guided diagnostic models.

Finally, the educational domain remains a cornerstone of prevention. Simulation-based modules can bridge theory and practice by incorporating virtual DAT-SPECT interpretation, medication review exercises, and case-based learning focused on deprescribing. López-Sendón et al. [3] emphasized that witnessing a patient’s recovery after discontinuing an offending drug reinforces diagnostic reasoning far more effectively than does didactic teaching alone. Embedding DIP cases within neurology clerkships, psychiatry rotations, and interprofessional training aligns with competency-based education frameworks that prioritize patient safety, interdisciplinary awareness, and the ability to recognize iatrogenic disease early. By linking mechanistic insight to real-world decision-making, education itself becomes a preventive tool against avoidable neurologic harm.

## Conclusions

DIP persists not because it is rare or obscure, but because it sits at the intersection of disciplines, such as neurology, psychiatry, geriatrics, and pharmacology, where communication often fails. This narrative review integrates epidemiologic, mechanistic, and educational perspectives to construct a mechanism-guided diagnostic framework that is both clinically actionable and pedagogically valuable.

By viewing DIP through this integrative lens, clinicians can transition from reactive recognition to proactive prevention. For medical educators, DIP embodies the principles of translational teaching: connecting receptor pharmacology to real-world patient care. For health systems, it exemplifies how surveillance data can be transformed into bedside vigilance. Ultimately, the most important lesson of DIP is humility: the reminder that not all parkinsonism is PD and that awareness, not technology, remains the first tool of diagnosis.
